# Supplementary material for: HIVprotI: an integrated web based platform for prediction and design of HIV proteins inhibitors
Source: J Cheminform. 2018 Mar 9;10:12. doi: 10.1186/s13321-018-0266-y (PMC5845081; doi:10.1186/s13321-018-0266-y)
Supplement: Supplementary file 2 — Additional file 2. Source code of HIVProtI web server. [file 13321_2018_266_MOESM2_ESM.zip › HIVprotI_Source-code/hivprotI/weka-3-6-6/doc/weka/classifiers/evaluation/EvaluationUtils.html]

EvaluationUtils


---


|  |  |  |
| --- | --- | --- |
| **Overview** \| **Package** \| **Class** \| **Tree** \| **Deprecated** \| **Index** \| **Help** \| **Weka's home** | |  |
| **PREV CLASS**   **NEXT CLASS** | **FRAMES**    **NO FRAMES**     **All Classes** |  |
| SUMMARY: NESTED \| FIELD \| CONSTR \| METHOD | DETAIL: FIELD \| CONSTR \| METHOD |  |


---


## weka.classifiers.evaluation Class EvaluationUtils

```
java.lang.Object
  weka.classifiers.evaluation.EvaluationUtils
```

**All Implemented Interfaces:**: RevisionHandler

---

``` public class EvaluationUtils extends java.lang.Object implements RevisionHandler ```

Contains utility functions for generating lists of predictions in
various manners.

**Version:**
:   $Revision: 1.11 $

**Author:**
:   Len Trigg (len@reeltwo.com)

---

**Constructor Summary**

`EvaluationUtils()`


| **Method Summary** | |
| --- | --- |
| `FastVector` | `getCVPredictions(Classifier classifier, Instances data, int numFolds)`             Generate a bunch of predictions ready for processing, by performing a cross-validation on the supplied dataset. |
| `Prediction` | `getPrediction(Classifier classifier, Instance test)`             Generate a single prediction for a test instance given the pre-trained classifier. |
| `java.lang.String` | `getRevision()`             Returns the revision string. |
| `int` | `getSeed()`             Gets the seed for randomization during cross-validation |
| `FastVector` | `getTestPredictions(Classifier classifier, Instances test)`             Generate a bunch of predictions ready for processing, by performing a evaluation on a test set assuming the classifier is already trained. |
| `FastVector` | `getTrainTestPredictions(Classifier classifier, Instances train, Instances test)`             Generate a bunch of predictions ready for processing, by performing a evaluation on a test set after training on the given training set. |
| `void` | `setSeed(int seed)`             Sets the seed for randomization during cross-validation |

**Methods inherited from class java.lang.Object**

`equals, getClass, hashCode, notify, notifyAll, toString, wait, wait, wait`

**Constructor Detail**

### EvaluationUtils

```
public EvaluationUtils()
```


**Method Detail**

### setSeed

```
public void setSeed(int seed)
```

:   Sets the seed for randomization during cross-validation

---


### getSeed

```
public int getSeed()
```

:   Gets the seed for randomization during cross-validation

---


### getCVPredictions

```
public FastVector getCVPredictions(Classifier classifier,
                                   Instances data,
                                   int numFolds)
                            throws java.lang.Exception
```

:   Generate a bunch of predictions ready for processing, by performing a
    cross-validation on the supplied dataset.

    :   **Parameters:**: `classifier` - the Classifier to evaluate: `data` - the dataset: `numFolds` - the number of folds in the cross-validation. **Throws:**: `java.lang.Exception` - if an error occurs

---


### getTrainTestPredictions

```
public FastVector getTrainTestPredictions(Classifier classifier,
                                          Instances train,
                                          Instances test)
                                   throws java.lang.Exception
```

:   Generate a bunch of predictions ready for processing, by performing a
    evaluation on a test set after training on the given training set.

    :   **Parameters:**: `classifier` - the Classifier to evaluate: `train` - the training dataset: `test` - the test dataset **Throws:**: `java.lang.Exception` - if an error occurs

---


### getTestPredictions

```
public FastVector getTestPredictions(Classifier classifier,
                                     Instances test)
                              throws java.lang.Exception
```

:   Generate a bunch of predictions ready for processing, by performing a
    evaluation on a test set assuming the classifier is already trained.

    :   **Parameters:**: `classifier` - the pre-trained Classifier to evaluate: `test` - the test dataset **Throws:**: `java.lang.Exception` - if an error occurs

---


### getPrediction

```
public Prediction getPrediction(Classifier classifier,
                                Instance test)
                         throws java.lang.Exception
```

:   Generate a single prediction for a test instance given the pre-trained
    classifier.

    :   **Parameters:**: `classifier` - the pre-trained Classifier to evaluate: `test` - the test instance **Throws:**: `java.lang.Exception` - if an error occurs

---


### getRevision

```
public java.lang.String getRevision()
```

:   Returns the revision string.

    :   **Specified by:**: `getRevision` in interface `RevisionHandler`
    :   **Returns:**: the revision


---


|  |  |  |
| --- | --- | --- |
| **Overview** \| **Package** \| **Class** \| **Tree** \| **Deprecated** \| **Index** \| **Help** \| **Weka's home** | |  |
| **PREV CLASS**   **NEXT CLASS** | **FRAMES**    **NO FRAMES**     **All Classes** |  |
| SUMMARY: NESTED \| FIELD \| CONSTR \| METHOD | DETAIL: FIELD \| CONSTR \| METHOD |  |


---
